# Supplementary material for: Learning curve and surgical outcome of robotic assisted colorectal surgery with ERAS program
Source: Sci Rep. 2022 Nov 29;12:20566. doi: 10.1038/s41598-022-24665-w (PMC9709162; doi:10.1038/s41598-022-24665-w)
Supplement: Supplementary file 2 — Supplementary Information 2. [file 41598_2022_24665_MOESM2_ESM.zip › RAL Raw data-Ñ[▒K/127/OP N_ñw╝╨░O▒Kñσ.pdf]

W55 / W55

臺中榮民總醫院
手術室護理記錄

[redacted]

基本資料

- 室別： 開刀房-17室，手術科別： CRS，手術類別： 常規，日期： 2021/11/18
- 進入時間： 1640 ； 麻醉開始時間： 1645 ； Time Out時間： 1750 ； 劃刀時間： 1751 ； 手術結束時間： 2140 送出時間： 2200 ；
- 麻醉方式： GE

  診斷： RO S COLON LIPOMA WITH INTUSSUSCEPTION

  手術名稱： Robot Assisted Colectomy(L)

  手術局部用藥： nil

  術前預防性抗生素：
  - 抗生素名稱一： Veterin 2GM IVD/由麻醉護理師給予
  - 時間： 1740
  - 上次簽章: 劉雅汶

  ，追加預防性抗生素：
- 手術醫師： 蔣鋒帆，陳瑢夫，蔡滄樟
- 麻醉醫師： VS 楊士杰
-
  - 刷手護士A ： 朱淨慧，時間： 1640，接替刷手一： 邱儷嫊，時間： 1700，接替刷手二： 朱淨慧，時間： 1730
  - 流動護士A ： 劉雅汶，時間： 1640，接替流動一： 邱儷嫊，時間： 1700，接替流動二： 劉雅汶，時間： 1730

皮膚狀況

A-V Shunt： 無

術前： 完整

記錄者： 上次簽章: 劉雅汶

術後： 不完整

部位(術後)：

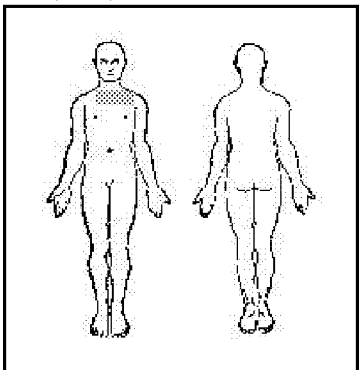

胸口30*10公分兩處壓紅不可反白、左胸前破皮

記錄者： 劉雅汶

手術準備

手術臥式 單一手術臥式

-
  - 手術臥式： 截石臥式
  - 使用附件： 溫毯 38 ℃，頭圈，腳架
  - 皮膚準備： 10%Aq Betadine ， Hibiscrub ， 2% Chlorhexidine
  - 電燒： 使用電燒: 電極片位置： 其它： 電燒迴路軟墊，雙極電燒，記錄者： 上次簽章: 劉雅汶

止血帶T： 無

植入物

無

標本送檢

- 細菌培養 無
- 細胞學 無
- 冰凍切片 無
- 病理標本
  組織名稱一： distal cut end
  ,
  組織名稱二： s colon
- 截肢 無

標本交付 標本： 1 袋 ， 標本： 0 盒 ， 標本： 1 瓶 ， 細胞學： 0 管 ， Culture Tube： 0 管 ， 被交付醫師： CR 陳瑲夫 ，

- 記錄者： 上次簽章: 劉雅汶
- 時間： 2100

傷口術後敷料

傷口敷料：

傷口敷料： 無

- 請於圖上標示部位：

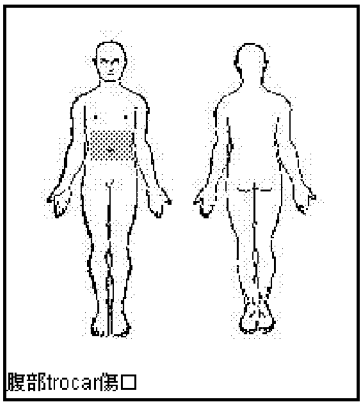

- 傷口敷料： 軟性敷料

導管

導管	天數	導管資訊	插入日期	插入時間	更換日期	拔除日期	拔除時間	拔除原因	預計拔除/更換	預計拔除/更換日期
Foley(1)	1	號碼:16 Fr.; 部位:膀胱; 註記:balloon 10c.c;	2021/11/18	1645						
Double Lumen CVC(1)	1	號碼:8 FR; 深度:15cm; 部位:subclavian (右); 象限:RU;	2021/11/18	1720						
Sump NG Tube(1)	1	號碼:16 Fr.; 部位:胃; 註記:fix 55cm;	2021/11/18	1730						
A-line (1)	1	號碼:20 G; 部位:左手;	2021/11/18	1650						
Epidural PCA(1)	1	部位:硬脊膜下;	2021/11/18	1700						

用物計數

- 手術中出血量： minimal c.c.
- 手術中尿量： 100 c.c.

用物計數： 多切口同時縫合

- 1.器械： 正確 ，2.縫針： 正確 ，3.刀片 ： 正確 ，4.紗布： 正確 ，5.腹部墊： 未使用 ，6.紗球： 未使用 ，7.PEANUTS ： 未使用 ，8.紗條： 未使用 ，9.絲帶： 未使用 ，10.針頭： 未使用 ，11.Vessel loop： 未使用 ，12.Boots： 未使用 ，13.Jelco： 未使用 ，14.棉條： 未使用 ，
  - 第一次：
    - 刷手護士 ： 朱淨慧
    - 流動護士： 上次簽章: 劉雅汶

        - 時間： 2050

,

- 第二次：
    - 刷手護士 ： 朱淨慧
    - 流動護士： 上次簽章: 劉雅汶
    - 時間： 2100

,

- 第三次：
    - 刷手護士 ： 朱淨慧
    - 流動護士： 上次簽章: 劉雅汶
    - 時間： 2120

備註

備註： NO.2 davinci 30度scope

- 記錄者： 上次簽章: 劉雅汶
- 時間： 1700

填寫護理人員 劉雅汶

正式摘要

W55 / W55

doctorRank:B
